# The LCMV gp33-specific memory T cell repertoire narrows with age

**DOI:** 10.1186/1742-4933-9-17

**Published:** 2012-08-15

**Authors:** Adam Bunztman, Benjamin G Vincent, Harsha Krovi, Shaun Steele, Jeffrey A Frelinger

**Affiliations:** 1 Department of Immunobiology, University of Arizona, Tucson, AZ, 85724, USA; 2 Departments of Medicine, University of North Carolina, Chapel Hill, NC, 27599, USA; 3 Microbiology and Immunology, University of North Carolina, Chapel Hill, NC, 27599, USA

**Keywords:** CD8 T cell, T cell repertoire, T cell receptor, Aging

## Abstract

**Background:**

The memory response to LCMV in mice persists for months to years with only a small decrease in the number of epitope specific CD8 T cells. This long persistence is associated with resistance to lethal LCMV disease. In contrast to studies focused on the number and surface phenotype of the memory cells, relatively little attention has been paid to the diversity of TCR usage in these cells. CD8^+^ T cell responses with only a few clones of identical specificity are believed to be relatively ineffective, presumably due to the relative ease of virus escape. Thus, a broad polyclonal response is associated with an effective anti-viral CD8^+^ T cell response.

**Results:**

In this paper we show that the primary CD8^+^ T cell response to the LCMV gp33-41 epitope is extremely diverse. Over time while the response remains robust in terms of the number of gp33-tetramer^+^ T cells, the diversity of the response becomes less so. Strikingly, by 26 months after infection the response is dominated by a small number TCRβ sequences. In addition, it is of note the gp33 specific CD8^+^ T cells sorted by high and low tetramer binding populations 15 and 22 months after infection. High and low tetramer binding cells had equivalent diversity and were dominated by a small number of clones regardless of the time tested. A similar restricted distribution was seen in NP396 specific CD8^+^ T cells 26 months after infection. The identical TCRVβ sequences were found in both the tetramer^hi^ and tetramer^lo^ binding populations. Finally, we saw no evidence of public clones in the gp33-specific response. No CDR3 sequences were found in more than one mouse.

**Conclusions:**

These data show that following LCMV infection the CD8^+^ gp33-specific CD8 T cell response becomes highly restricted with enormous narrowing of the diversity. This narrowing of the repertoire could contribute to the progressively ineffective immune response seen in aging.

## Background

The cell mediated immune response is critical in the clearance of many viral infections. Lymphocytic choriomeningitis virus (LCMV) is one the most widely studied acute viral diseases in experimental animals [1,2]. For LCMV clearance there are critical roles for both CD4^+^ and CD8^+^ T cells, but it is clear that CD8^+^ memory cells are vitally important for the resistance to secondary challenge [3,4]. In LCMV infections there are three distinct phases of the CD8 T cell responses: priming, expansion and contraction [4]. Following virus clearance, antigen specific CD8^+^ T cells persist as memory cells for many months- essentially the lifetime of the mouse [5] and the persistence of T cells may or may not depend of signaling through TCR depending on the specificity of the T cell [6]. Intensive work has shown that the number of antigen specific CD8^+^ T cells in mice declined only slowly over time. In mice the half life for tetramer^+^ CD8^+^ T cells in the spleen was nearly 2 years [7]. This long lifespan has been seen in many virus specific T cell populations in both mouse and man [8-10].

In contrast, the body of work enumerating the number of LCMV specific CD8 T cells, the T cell receptor diversity of those cells has been investigated only sporadically. Lin and Welsh examined the total TRVβ13-3 (IMGT nomenclature is used throughout, older nomenclature is translated to IMGT) repertoire by spectrotyping [11]. They concluded that the repertoire changed little after virus clearance, although superinfection with an unrelated virus did change the LCMV specific repertoire significantly [12]. Similarly, Blattman et al. found little change between the primary and secondary responses in terms of TCR repertoire, but their characterization was also limited to spectratyping [13]. Others have found a similar large diversity of LCMV specific clones following tetramer sorting after acute LCMV infection [14].

In aging, humans and mice often display an accumulation of a single T cell clone that might occupy as much as 30% of the total CD8^+^ T cells [15-18]. This is known as T cell clonal expansion (TCE). T cell expansions have a memory phenotype and are widely believed to arise from existing memory cells. These TCE are apparently inherently unstable and have a variable phenotype [19-23]. While there has been significant interest in these cells and their function, there has been relatively little work performed to link the TCE to virus specific T cells.

Relatively little work exists concerning the overall TCRβ diversity of the virus specific responses measured directly ex vivo. Much of the data involves the use of either T cell cloning or spectratyping to evaluate the overall repertoire. In the case of spectrotyping, these results can both under and over-estimate the diversity. T cell cloning is plagued by strong selection for cells able to grow in vitro. Much of the literature on TCE suggests that these cells grow poorly in vitro and so would be under counted in experiments that require growing T cells.

In this study we have examined the repertoires of CD8 T cells specific for the LCMV epitope gp33 immediately following infection and more than two years later, The epitope specific T cells slowly declined in numbers as expected. The initial response was highly diverse with an essentially a flat distribution and no clone representing more than 3% of the total epitope specific CD8^+^ T cells. While the fraction of epitope specific cells was nearly constant, the diversity was dramatically restricted with age. When the mice were tested at 15 months following infection we found that the diversity had decreased with 17% of the gp33^+^ response represented by a single Vβ; by 26 months 100% of the gp33-specific TCRβ sequences were a single TCRβ clone in one of the mice. Only 13 unique sequences were found in the other 26 month old mouse. This strongly supports the idea that clonal expansions do not arise from a distinct lineage, but from the regular memory pool as has been previously proposed [16,24].

## Results

We report a total 1143 Vβ sequences from individual CD8^+^ T cells isolated from five mice at varying times before and after LCMV infection. We evaluated a single mouse at 23 days, 15 and 22 months after infection. Two mice were tested 26 months after infection. We recovered 375 unique TCRβ sequences specific for gp33 specific CD8^+^ T cells. We have deposited all sequences in GenBank (Accession numbers JX277204 – JX277543) and summarized the results in Additional file 1: Table S1.

### Vβ Usage

**Figure 1:**
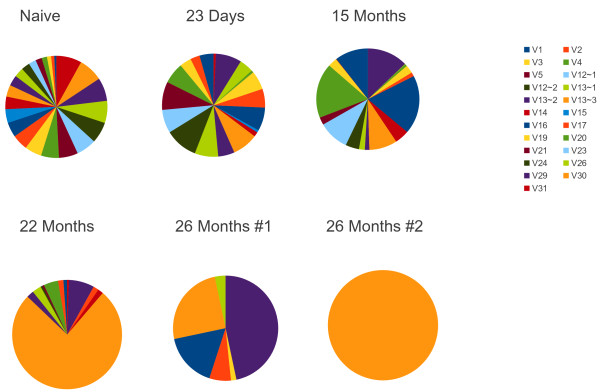
**Pinwheel depiction of the TCRVβ usage of gp33-tetramer**^**+ **^**cells following infection with LCMV. Naive represents the unimmunized repertoire.** Each pinwheel represents the distribution of TCRVβ from tetramer sorted cells from a single mouse. Two mice were tested at 26 months, designated #1 and #2. The number of cells sequenced in each pinwheel is: naïve, 55; 23 Days, 136; 15 months, 705; 22 months, 187; months 26 mouse 1, 60; 26 months mouse 2, 64. The entire CDR3 sequences have been deposited in Genbank, Accession numbers: [Genbank:JX277204 – JX277543].

We report here 55 randomly sequenced TCRβ sequences. In addition we have sequenced 120,000 TCR Vβ from unselected B6 splenic T cells (Buntzman, Krovi and Frelinger, unpublished). These sequences have a similar distribution of Vβ usage consistent with previous work by others (reviewed in [25]). The Vβ usage in the 55 single cells is reported here and is shown in Figure 1. When we sequenced TCRβ from gp33 sorted CD8^+^ T cells 23 days following infection with LCMV, we found 132 different sequences (Additional file 1: Table S1). Eight per cent were TRVβ13-3 (Figure 1). However we also found 10% TRVβ12-2 and nearly equivalent numbers of TRVβ3, TRVβ12-1 TRVβ16 and TRVβ30. We compared the pattern of Vβ usage between the naïve and 23 day post infection V gene usage and found a significant correlation (p < .003) demonstrating that the initial gp33 specific repertoire is large and representative of the overall diversity. 

### Entropy Decline

**Figure 2:**
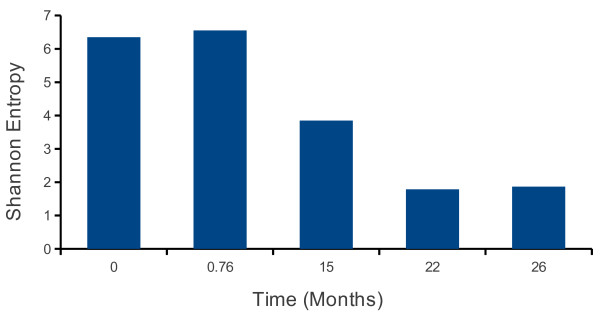
**Shannon Entropy of the TCRVβ usage of gp33 tetramer**^**+ **^**CD8 T cells over time following infection with LCMV.** Shannon entropy was calculated for each distribution using the pooled data from tetramer high and low cells at 15 and 22 months. The entropy was calculated separately for the 26 month mice and averaged.

Entropy has been used by ecologists to describe the combined species richness (the number of species present) and the distribution of species present (the percentages of the total individuals of each species) [26,27]. This number is analogous to chemical entropy as it measure the total disorder in a system. Thus a population with more species has a higher entropy, as does one with a flatter distribution of the number of individuals of each species. We have chosen to use the Shannon entropy as the index of diversity that accounts for both the number and distribution of species as previously described by our group (41). The entropy is calculated from the sequence data- that is the number and distribution of VJ and CDR3. When we compared the entropy between the day 23 and naïve T cells, both had similar, large entropy (6.4 compared to 6.5) (Figure 2), consistent with high species richness and the lack of dominant clones. Based on the spectratyping analysis of B6 mice we had expected the number of T cell receptor CDR3 to be relatively restricted [13]. Instead we found TRVβ13 genes were used as predicted although they represented only a small fraction (8%) of the gp33 response. This indicates that spectratyping can significantly underestimate the diversity of the T cell receptors used. We emphasize that these sequences came from tetramer sorted single cells. 

We sequenced TCR from gp33^+^ T cells from the spleens of mice 15 months after infection. By this time, the response had significantly fewer unique TCRβ represented (Figure 1), We found only 141 different clones among the 705 TCR sequenced. Further, there was a narrowing of the Vβ usage, with TRVβ16 present in the highest frequency (18.7% followed by TRVβ4, 17.4%; TRVβ1, 10.5%; TRVβ12, 9.6% and TRVβ13-3, 12.7%). Together, these make up 67% of the sequences recovered. At 15 months post infection the entropy had decreased from 6.5 to 3.8, a large decrease in diversity (Figure 2). There was no significant correlation of TRVβ usage with the 23 day post infection repertoire, consistent with the narrowing of the responsive repertoire as measured by the large decrease in entropy. There was also no significant correlation with the naïve repertoire Vβ usage.

We performed an identical experiment sorting gp33^+^ T cells at 22 months post infection. Here we recovered 187 Vβ sequences. In this mouse 75% of the Vβ sequences were TRVβ13-3, with 8% TRVβ29. Of the 142 TRVβ13-3 sequences recovered, there were only two unique sequences represented. This was a marked change from the 15 month sample. As would be expected from the clonal dominance, the entropy had further decreased to 1.8. There was a significant correlation with the Vβ usage with the 15 month sample as judged by Pearson's correlation (p < .001).

In the sample from two mice 26 months after infection, the Vβ usage had decreased even further. One of the mice used only a single Vβ and CDR3. The other mouse used only 6 Vβ with TRVβ29, TRVβ13-3 and TRVβ16 making up the majority of the cells (Figure 1). Using only the data from the more diverse mouse, the entropy had decreased still further (Figure 2).

**Figure 3:**
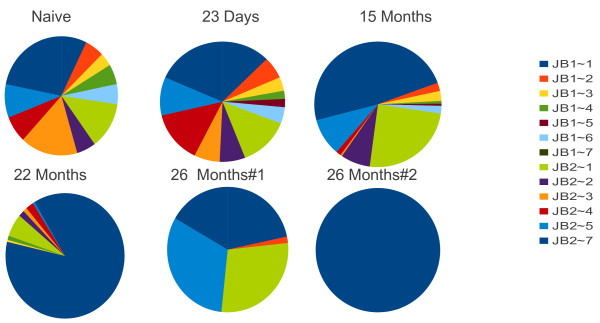
**Pinwheel depiction of the TCR Jβ usage of gp33-tetramer**^**+ **^**cells following infection with LCMV.** Naive represents the unimmunized repertoire. The distributions are derived from the same sequences described in Figure 1.

We performed an identical analysis on Jβ usage (Figure 3). Using the same approaches we found the same results- Jβ usage decreased as a function of time after infection and the same conclusion is reached- there is a sequential enrichment of a relatively small number of clones.

### Vβ Jβ pair usage narrows over time

**Figure 4:**
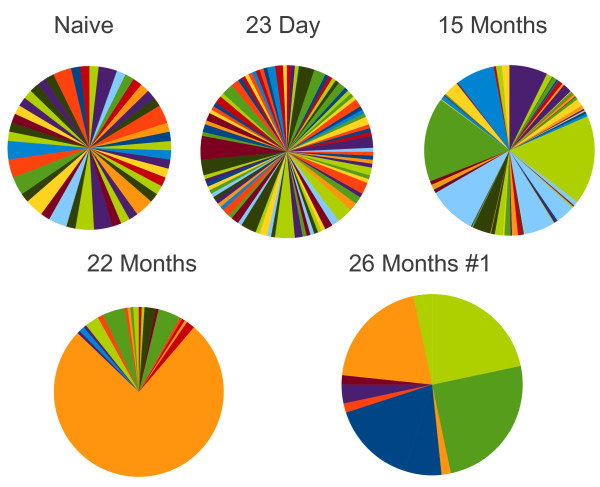
**Pinwheel depiction of the VβJβ pairs used in gp33-tetramer**^**+ **^**cells following infection with LCMV.** Naive represents the unimmunized repertoire. The distributions are derived from the same sequences described in Figure 1. The legend is not shown because it is impossible to display all 284 VJ combinations.

Using the specific combination of Vβ Jβ pairs, Figure 4 shows the striking narrowing of the repertoire. We show the pinwheel of the initial Vβ Jβ pairs after initial infection, but by 15 months the pattern had strikingly simplified. At 26 months post infection just four Vβ Jβ combinations represented 82% of the clones, TRVβ2-/Jβ 2-1, 25%; TRVβ29/Jβ 2-5, 22%; TRVβ13-3/Jβ 1-1, 20% and TRVβ16/Jβ 2-5 15%. In a second 26 month post infection mouse 100% of the gp33-specific T cells were TRVβ13-3/Jβ 2-7. Thus, the dominance of the TRVβ13-3 T cells varies from mouse to mouse, but the narrowing of the repertoire did not.

Strikingly, no TCR CDR3 were shared among any of the these mice. Here we found no evidence of public phenotypes in the gp33-specific D^b^ restricted response.

### Slow decline in gp33 specific T cells

**Figure 5:**
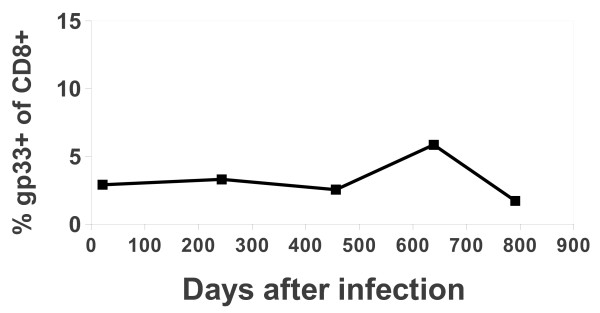
**Stability of the fraction of gp33 tetramer**^**+ **^**T cells over time following LCMV infection.** Here we show the fraction of CD8^+^ cells that were stained with the LCMV-D^b^-gp33 tetramer at each time. Data shown is from the primary sort files, except for the 23 days sames which comes from a separate experiment and is an average of 3 mice. Pearson correlation coefficient shows no significant correlation with time.

The number of gp33^+^ CD8^+^ T cells decreased slowly over time as has been previously reported [28]. However, the decrease was relatively small in our experiment; decreasing from 3% of CD8^+^ (measured in a separate experiment) to approximately 1.7% over the two year period of observation (Figure 5). Thus there is a two fold decrease over this time period. The loss of heterogeneity cannot be accounted for simply by the selective loss of gp33^+^ CD8^+^ T cells since the frequency of gp33^+^ T cells remains high. This is similar to the frequency of loss of virus specific T cells previously reported [5,7-10]. 

### Vβ sequences are shared in high and low tetramer binding CD8^+^ T cells

**Figure 6:**
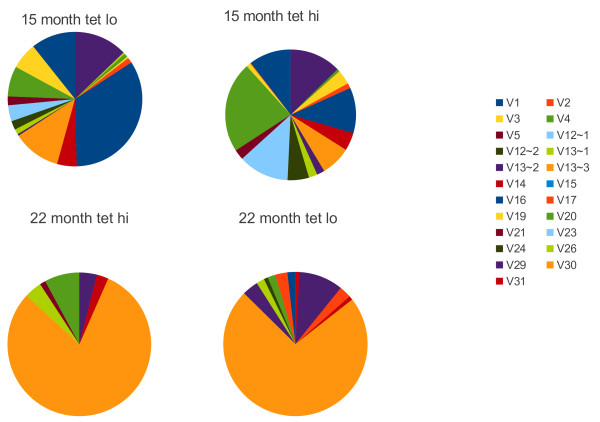
**Pinwheel depiction of the TCRVβ usage of gp33-tetramer**^**hi **^**cells and gp33-tetramer**^**lo **^**15 and 22 months following infection with LCMV.** Cells were sorted into D^b^-gp33 tetramer high binding and low binding populations. Single cells were sequenced and each pinwheel describes the distribution of TCRVβ expression. 15 month tetramer^hi^ binding is represented by 471 cells, and tetramer^lo^ by 234 cells; 22 month tetramer^hi^ by 76 and tet^lo^ by 111 cells.

We sorted gp33^+^ T cells from the 15 month post infection spleens into high and low tetramer binding populations. We compared the TCRβ sequences from gp33^hi^ tetramer binders with the gp33^lo^ binders. The amount of tetramer bound is often used to estimate the affinity of the TCR in cells, with higher binding thought to represent high affinity T cells. When we analyzed those populations separately we found a very similar distribution of Vβ usage as well as a number of identical sequences in both the 15 month tetramer^hi^ and tetramer^lo^ samples (Figure 6). Twenty-nine Vβ sequences were shared between the two populations. Since each sequence is derived from a single cell, the duplication of sequences cannot be attributed to sequencing artifacts. Indeed, one sequence appears 42 and 75 times in the high and low binding populations respectively.

**Figure 7:**
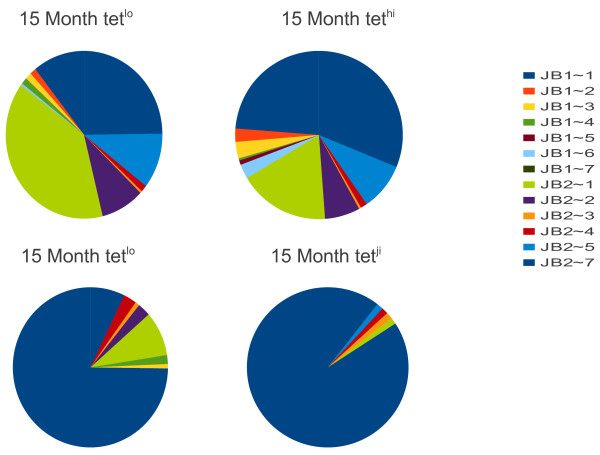
**Pinwheel depiction of the TCR Jβ usage of gp33-tetramer**^**hi **^**cells and gp33-tetramer**^**lo **^**15 and 22 months following infection with LCMV.** The distributions are derived from the same sequences described in Figure 4.

We performed a similar experiment with a 22 month post infection mouse. We sorted gp33^+^ CD8^+^ T cells into high and low binding populations and found that there was little difference between the high and low tetramer binding cells. Both were dominated by the same TRVβ13-3 sequence and both used Jβ 1-1 with one sequence present 61 and 81 times in the tetramer high and low populations respectively (Figure 6). When we examined the Jβ usage in tetramer^hi^ and tetramer^lo^ binding cells we found the same result (Figure 7) with there being no significant difference in Jβ usage in tetramer^hi^ and tetramer^lo^ cells. While it is possible that the shared sequences express different Vα chains it seems unlikely that all of the differences in tetramer binding would be due to differences in affinity mediated by Vα.

### NP396 specific CD8^+^ T cells also have a restricted repertoire in old mice

**Figure 8:**
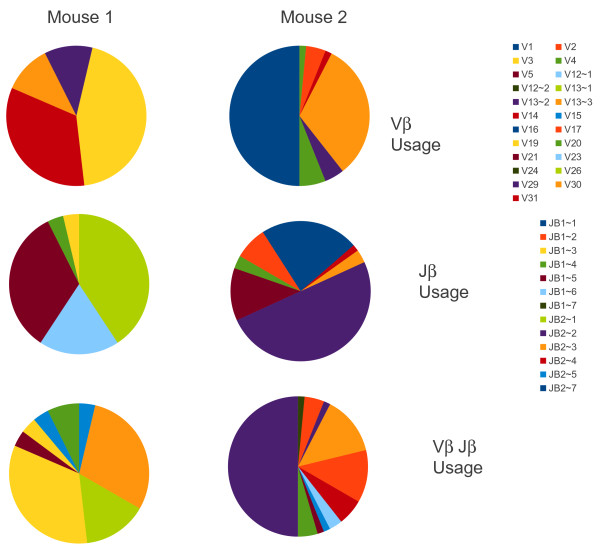
**Pinwheel depiction of TCR usage of NP396 specific CD8**^**+ **^**T cells sorted from 26 month post LCMV infection mice.** The NP396 cells are sorted from the same mice in the previous experiments. Mouse 1 is represented by 27 sequences and mouse 2 by 66 sequences.

To determine if this phenomenon was limited to gp33 specific T cells we sequenced NP396 specific T CD8^+^ T cells at 26 months in the same mice that were donors for the gp33-specific T cells. Figure 8 shows the usage of TRVβ, Jβ and Vβ/ Jβ pairs in these mice. As we saw with the gp33 specific T cells, the NP396 specific T cells also had a restricted repertoire compared to the naïve cells (Figure 1 compared to Figure 8). These NP396 tetramer^+^ T cells represented 2.5% and 0.6% of the CD8^+^ T cells from 2 mice, so their frequency was similar to that of the gp33 specific T cells. The calculated entropies of 3.4 and 2.5, is very similar to those found in the gp33 tetramer^+^ CD8^+^ T cells. Thus, although we do not have data from earlier time points for the NP396 specific T cells, in old mice the memory repertoire was similarly contracted to the gp33 specific repertoire.

Together these data require that some TCRβ increase at the expense of the other LCMV specific T cells recognizing the same epitope. This is not a simple survival advantage, but must include a proliferative advantage because the loss of the other clonotypes is not sufficient to account for the increase in the dominant clones. The data presented here not speak to any mechanism for the decreased variability, but could result from fortuitous expression higher expression of receptors for IL-5 or IL-15 that are completely unrelated to the T cell receptor. Further, although TRVβ13-3 was always an important component of the response, its dominance varied from 100% down to 20%.

## Discussion

As mammals age, they become increasingly unable to mount de novo immune responses [21,29,30]. Surprisingly, even pre-existing immune responses may also be impaired [31]. There are three broad, nonexclusive explanations for this. First, antigen specific cells might be absent; second, the antigen specific clones might be present, but unable to respond, and finally, the specificity of the clones might be off target as in the case of dengue fever [32]. The data we and others have found in old mice suggests that at least a substantial frequency of CD8^+^ memory T cells are present that are able to bind MHC/peptide complexes in the form of tetramers [29]. Therefore it seems unlikely that the first explanation is correct. However, the fact that many of the clones express a common TCRβ chain hints that the clones might have a restricted specificity, rather than being anergized.

The initial response to gp33 is very diverse. As a result, the Vβ usage is significantly correlated with the naïve repertoire. The correlation of the LCVM specific repertoire with the naïve repertoire is not surprising considering its high entropy. Vβ usage in the 15 month repertoire is not significantly correlated with the naive repertoire, although there is a significant correlation with the day 23 sequences. This is consistent with the continuous narrowing of the response, where each new repertoire measurement would be a subset of the previous one but with most of the clones disappearing over time. The observed restriction was most pronounced in the 26 month post infection samples where in one mouse we observed 4 dominant Vβ sequences, and in another we saw a single Vβ sequence. To establish this definitively, sequential sequence analysis of the repertoire within a single mouse would need to be followed over time.

We also analyzed the Vβ Jβ pairs. This analysis is substantially less robust due to the large number of pairs (284) of Vβ Jβ combinations. In this analysis we found no clear pattern as we did in the Vβ usage and showed no significant correlations by Pearson's test.

There are many reports concerning TCE in both mice and humans [15-20,22,23,33,34]. There is no clear consensus concerning the genesis of TCE except the expansions are not malignant cells and are not associated with any obvious pathology. Substantial speculation exists as to the source of the expanded T cells. The data we present here is completely consistent with TCE being derived from the normal T memory pool. In the two old mice we examined, it is possible that both would be detected as TCE, although the expansion in mouse 1 might be below the level of detection. For mouse 2 the frequency of gp33-specific T cells was still over 3% and there was essentially only a single gp33 clonotype detected. Even in bulk sequencing of TCRβ from this mouse an expanded clonotype of this size would be easily detected as an expansion of the TRVβ13-1 family. We speculate that TCE are the result of a predictable narrowing of the repertoire of a highly immunogenic challenge to the immune system.

It is tempting to speculate about the biological impact of such a narrow gp33 response. We know that infection of a monoclonal gp33 P14 mouse with high titer virus stock results in the selection of escape mutants [35,36]. We might predict that a similar event would happen if these mice were rechallanged with LCMV. However, other anti-LCMV [37] specific T cells are present. Memory responses to NP396 are well documented following LCMV infection. We observed a high frequency of NP396 specific cells even after 26 months. Thus in vivo, while there might be escape mutants favored for a specific epitope for effective virus persistence, the virus would need to nearly simultaneously mutate multiple epitopes at once, which seems unlikely.

The mechanism by which such expansion of CD8 T cells would arise is not clear. Many have speculated that high avidity clones are selected for following virus infections [38,39]. This seems logical based on the well documented affinity maturation of antibody responses [40]. Using cell sorting, we were able to address this notion. When cells were sorted based on tetramer binding into high and low tetramer binding cells we could compare the complexity of the repertoire Vβ usage in the high and low tetramer binding population. These populations were highly correlated in both the 15 and 22 month samples, suggesting that the tetramer binding does not effectively discriminate these two populations. This is in accordance with our published work that shows the tetramer binding of the identical TCR is dependent on the level of CD8 expression [41].

While we have collected much less data on the response of these same mice to the NP396 epitope, we find 20 unique sequences among 93 cells sequenced with a calculated entropy of 2.58 that is only slightly more complex than the entropy calculated for the pooled gp33 mice (1.86) but enormously less for the total repertoire in a naïve mouse (6.3). Thus it seems likely that a similar contraction of the epitope specific repertoire occurs in both the gp33 and NP396 populations.

We are not the first to study the LCMV specific T cell repertoire. Lin and Welsh examined the long and short term memory response of mice to LCMV by limited sequencing and spectratyping focusing on Vβ13-3 [11]. There they found a relatively stable pattern in the memory pool as long as 7 months following infection. When we examined the entropy of mice as young as 15 months post infection we saw a significant decrease in the entropy. It is possible the Welsh's group did not detect the contraction by 7 months due to focusing on TRVβ13-3. Alternatively, it might be that the the repertoire is stable for seven months and only begins to contract after that time. Our data do not allow us to rule out that possibility that the decline in repertoire complexity is not linear. This same group saw a profound narrowing of the LCMV repertoire as a result of infection with another virus [42]. While we cannot completely exclude the possibility that our mice became infected with a second virus, we find this extremely unlikely as our mice were housed in pathogen-free conditions prior to and following LCMV infection.

## Conclusions

Our work demonstrates that the diversity of a memory T cell receptor repertoire can progressively decrease over time in the absence of persistent antigenic stimulation. This may result from a survival "program" of particular clonotypes determined by TCR sequence at the time of the initial immune response and clonal expansion, or may represent stochastic success of expanded clonotypes independent of their TCR.

## Materials and Methods

### Mice

C57Bl/6 J (B6) mice were purchased from Jackson labs and housed in an AALAC accredited, SPF facility at UNC. Previously infected mice were maintained under BSL2 conditions, All procedures were approved by the UNC IACUC.

### LCMV Infection

Mice were infected by intraperitoneal injection of 10^4^ pfc of Armstrong 3 LCMV at 6 weeks of age. A single cohort of 5 mice was injected and followed over time. Mice were sacrificed and their LCMV specific repertoire was analyzed as below.

### Purification of LCMV specific CD8^+^ memory cells

Spleen cells were prepared from mice 23 days, 15, 22 and 26 months after infection. Tetramers were assembled from D^b^ protein produced in E. coli and stored as inclusion bodies before refolding. Proteins were refolded in vitro with either gp33 (KAVYNFATM) or NP 396 (FQPQNGQF) peptide. Purified monomers biotinylated in vitro with BirA and were purified by size exclusion chromatography and assembled with PE labeled ultra-Avidin (deglycosylated avidin). LCMV gp-33 or NP-396 specific CD8^+^ T cells were purified by flow cytometry using D^b^-gp33 or NP specific tetramers as previously described [43]. Lymphocytes were stained with anti CD8, tetramer, anti CD19 and anti CD4. CD8^+^ tetramer^+^ CD19^-^, CD4- cells were sorted using a MoFlo XDP cell sorter at the UNC flow cytometry faculty. Cells were sorted at one cell per well into 96 well plates.

### TCR sequencing and analysis

Cells were sorted at one cell per well into 96 well plates directly into trizol. 4μL of an osmotic lysis buffer (2μL PBS, 2μL nuclease-free water with 10 mM DTT and 10U RNAseIN). Plates were immediately flash-frozen on dry ice and stored at −80°C until rt/pcr amplification. Sequences were determined following RT/PCR using a degenerate primer set that amplifies all TCRβ chains and cycling conditions as previously reported in Vincent et al. [41]). TCR were amplified and sequenced as previously described [41].

### Statistical analysis

Statistical analysis was carried out using SOFA version 1.1.4 (Paton-Simpson & Associates Ltd, Auckland, New Zealand). P values for Pearson's correlations were corrected for multiple comparison using Bonferroni correction. Shannon Entropy was calculated using software developed and supplied by T. Kepler (Boston University) as we previously described [44].

### Nomenclature

We have consistently used the current IMGT nomenclature in this manuscript [45].

## Abbreviations

TCE: T cell expansion; LCMV: Lymphocytic choriomeningitis virus.

## Competing interests

The authors declare no competing interests.

## Authors contributions

AB, BV and SS carried out the cell purification and the DNA sequencing. AB, BV and HK carried out the bioinformatics analyses. JAF conceived of the project and drafted the manuscript. All authors revised the manuscript and analyses and approved of the manuscript.

## Supplementary Material

Additional file 1 **Table 1.** Summary of Sequence Data. Click here for file
